# Towards an anti-fibrotic therapy for scleroderma: targeting myofibroblast differentiation and recruitment

**DOI:** 10.1186/1755-1536-3-8

**Published:** 2010-05-27

**Authors:** Andrew Leask

**Affiliations:** 1 Division of Oral Biology, Department of Dentistry, Schulich School of Medicine and Dentistry University of Western Ontario, Dental Sciences Building, London ON N6A 5C1 Canada

## Abstract

**Background:**

In response to normal tissue injury, fibroblasts migrate into the wound where they synthesize and remodel new extracellular matrix. The fibroblast responsible for this process is called the myofibroblast, which expresses the highly contractile protein α-smooth muscle actin (α-SMA). In normal tissue repair, the myofibroblast disappears. Conversely, abnormal myofibroblast persistence is a key feature of fibrotic dieases, including scleroderma (systemic sclerosis, SSc). Myofibroblasts can be derived from differentiation of local resident fibroblasts or by recruitment of microvascular pericytes.

**Clinical problem addressed:**

Controlling myofibroblast differentiation and persistence is crucial for developing anti-fibrotic therapies targeting SSc.

**Basic science advances:**

Insights have been recently generated into how the proteins transforming growth factor β (TGFβ), endothelin-1 (ET-1), connective tissue growth factor (CCN2/CTGF) and platelet derived growth factor (PDGF) contribute to myofibroblast differentiation and pericyte recruitment in general and to the persistent myofibroblast phenotype of lesional SSc fibroblast, specifically.

**Relevance to clinical care:**

This minireview summarizes recent findings pertinent to the origin of myofibroblasts in SSc and how this knowledge might be used to control the fibrosis in this disease.

**Conclusions:**

TGFβ, ET-1, CCN2 and PDGF are likely to cooperate in driving tissue repair and fibrogenic responses in fibroblasts. TGFβ, ET-1 and CCN2 appear to contribute to myofibroblast differentiation; PDGF appears to be involved with pericyte recruitment. Thus, different therapeutic strategies may exist for targeting the multisystem fibrotic disorder SSc.

## Introduction

**Figure 1:**
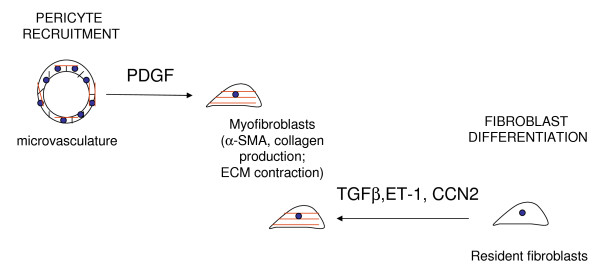
**Model of myofibroblast origin in skin lesions of scleroderma patients**. Transforming growth factor β, endothelin-1 and CCN2 can enhance differentiation of resident fibroblasts to myofibroblasts; platelet derived growth factor promotes pericyte recruitment.

When connective tissue is damaged, fibroblasts migrate into the wound and begin to produce and remodel extracellular matrix (ECM) [1]. These events involve a specific sort of fibroblast termed the myofibroblast, a cell type which expresses the highly contractile protein α-smooth muscle actin (α-SMA) [1]. The α-SMA protein is organized into stress fibres which are connected to the ECM through specialized so-called 'supermature' FAs. As a result, these α-SMA stress fibers can contract and exert mechanical tension on the ECM causing it to be reorganized into functional connective tissue. Myofibroblast persistence is believed to be responsible for fibrotic diseases including scleroderma (SSc; Figure 1) [1,2].

Myofibroblasts have multiple origins, possibly appearing, for example, by differentiation of local, resident fibroblasts in response to proteins or by the migration of microvascular pericytes into the lesional area [1] (Figure 1). Understanding how myofibroblasts may originate may be useful in understanding how to combat the fibrosis observed in SSc, and this is the subject of this minireview.

### Transforming growth factor-β (TGF-β)

Extensive reviews on TGFβ signalling and the contribution of this pathway to experimentally-induced fibrosis have been published elsewhere (for example, see [3]). Briefly, there are three TGFβ isoforms, TGFβ1, TGFβ2 and TGFβ3. These are initially generated as latent precursors from which active TGFβ is liberated by proteolysis, enabling it to bind to a heteromeric receptor complex consisting of one TGFβ type I [termed activin linked kinase 5 (ALK5) in the case of fibroblasts] and one TGFβ type II receptor. ALK5 phosphorylates Smad2 and 3, which can then bind Smad4, translocates into the nucleus and activate transcription. The ALK5/Smad pathway is generally responsible for TGFβ signalling in fibroblasts. In normal fibroblasts, ALK5 appears to mediate the fibrogenic activity of TGFβ [4]. Recombinant TGFβ is fibrogenic in both *in vitro *and *in vivo *models of fibrogenesis, acting through ALK5/Smad3 [3]. The contribution of this canonical TGFβ pathway on the persistent fibrotic phenotype of lesional SSc fibroblasts has been evaluated. Targeting ALK5, using small molecule inhibitors, reverses some aspects of lesional dermal scleroderma fibroblasts but, critically, does not reduce α-SMA or CCN2 protein expression or α-SMA stress fibre formation in this cell type [2,5,6]. These results are consistent with data showing that Smad3 is not involved with CCN2 overexpression in SSc fibroblasts [7]. These observations exist in spite of the fact that there is a trend towards heightened Smad3 phosphorylation and nuclear localization in SSc fibroblasts compared to their healthy counterparts [8].

An interesting series of recent experiments have suggested the possibility that heightened activity of Smad1 may contribute to the pathogenesis of SSc [9]. This phenomenon appears to arise due to an alteration in the ratio of the levels of the TGFβ type I receptor to the TGFβ type II receptor. In SSc fibroblasts, there is an elevation in the amount of the TGFβ type I receptor which may contribute to the overexpression of type I collagen in these cells [10]. Overexpression of ALK5 caused an increase in collagen production by fibroblasts [10,11]. This up-regulation of collagen does not appear to involve the kinase activity of ALK5 or Smad2/3 activation but experiments using mutant TGFβ receptors and siRNAs show that this phenonomenon appears to be mediated by ALK1/Smad1 and ERK1/2 pathways [9]. Overall, these data suggest that canonical ALK5/Smad signalling is likely to contribute to but not be the fundamental basis of the persistent fibrotic phenotype of lesional SSc fibroblasts and suggest that blocking non-canonical TGFβ pathways may be a better alternative in combating the persistent fibrotic phenotype in SSc [12,13].

In addition to the ALK5/Smad pathway, TGFβ activates additional signalling pathways including: ras/MEK/ERK, which requires the heparan sulphate-containing proteoglycan (HSPG) syndecan 4; p38, which requires the HSPG betaglycan and JNK which requires focal adhesion kinase (FAK) and TGFβ activated kinase 1 (TAK1) [2,14,15]. These pathways appear to modify gene expression in a promoter-selective fashion. For example, FAK, JNK and TAK1 are required for myofibroblast differentiation and α-SMA expression [14,15]. Whereas extra cellular signal-regulated kinase (ERK) is required for CCN2 and collagen type I expression [16,17], p38 appears to be not involved with the fibrogenic activity of TGFβ [4]. Constitutive TAK1 and JNK activation independent of ALK5 is seen in SSc fibroblasts [14,18]; thus, it is likely that signalling pathways are abnormally activated in SSc fibroblasts in a fashion independent of the canonical TGFβ pathway. It is likely that targeting FAK, JNK or TAK1 may be beneficial in alleviating the persistent SSc phenotype of dermal fibroblasts.

### Endothelin-1 (ET-1)

There are 3 isoforms of endothelin, namely ET-1, ET-2, and ET-3 [12]. ET-1, the significant isoform in humans, is normally produced by a variety of cell types including endothelial cells, epithelial cells, bone marrow mast cells, macrophages, polymorphonuclear leukocytes, cardiomyocytes, and fibroblasts [12]. Initially, ET-1 is produced in the form of a 212-amino acid precursor (prepro-ET-1) which is enzymatically cleaved to form a biologically active 21-amino acid peptide [12]. ET-1 can then bind its two receptors (ET_A _and ET_B_) [12].

ET-1 induces ECM production in fibroblasts through the ET_A _and ET_B _receptors and MEK/ERK, whereas ET-1 induces myofibroblast formation, migration and ECM contraction through ET_A _and Akt/rac [19,20] (Figure 1). TGFβ induces ET-1 through JNK, and ET-1 is a downstream mediator of at least some of fibrotic responses of fibroblasts to TGFβ [18,21]. Constitutive ET signalling, operating through TAK1/JNK-dependent and ALK5-independent mechanisms, is responsible for the persistent myofibroblast phenotype of SSc lung fibroblasts [18]. Consistent with the notion that ET-1 contributes to fibrosis in the lung, ET receptor antagonism alleviates bleomycin-induced lung fibrosis and TGFβ-induced skin fibrogenesis *in vivo *[22,23]. However, the effect of ET inhibition on SSc dermal fibroblasts has not yet been tested. TGFβ appears to also cooperate with ET-1 to promote myofibroblast differentiation [24]. The ET receptor antagonist bosentan may also be effective at reducing skin fibrosis in patients with SSc [25]. These results suggest that endothelin receptor antagonism might be considered as an appropriate therapy for the fibrosis in SSc, possibly in combination with anti-TGFβ regimens.

### CCN2

CCN2, a member of the CCN family of matricellular proteins, is an excellent surrogate marker for the severity of fibrosis in SSc [26]. CCN2 signals through a variety of integrins and HSPGs or trkA and promotes cell adhesion and enhances adhesive signaling in response to extracellular ligands [27]. CCN2 is induced by both TGFβ and ET-1 and is considered to be a downstream mediator of these proteins [12]. The CCN2 promoter appears to possess independent TGFβ and ET-1 response elements [16,20] and thus may be a common downstream mediator of the fibrotic effects of these proteins, and thus may represent a more attractive target than either protein alone.

CCN2 acts as a cofactor with TGFβ to induce fibrogenesis but is not considered to be a potent fibrogenic agent on its own [28,29] (Figure 1). However, a recent study revealed chronic overexpression of CCN2 can lead to a fibrotic phenotype [30]. CCN2 is not required for all of the activities of TGFβ actions but appears to be required for TGFβ to maximally induce certain mRNAs including type I collagen and α-SMA and for TGFβ to promote cell adhesion to ECM [31] (Figure 1). CCN2 also can activate ERK by a syndecan 4-dependent mechanism [32]. A CCN2 response element exists in the COL1A2 promoter; blocking CCN2 action using an anti-CCN2 antibody or siRNA reduces some effects of bleomycin-induced lung fibrosis [33]. Overall, the available data suggest that targeting CCN2 may be useful in combating fibrosis in SSc.

### Platelet derived growth factor (PDGF)

The PDGF family includes PDGF-AA, PDGF-AB, PDGF-BB, PDGF-CC and PDGF-DD. These bind two different PDGF receptors, α and β [34]. PDGF causes neutrophils, macrophages, fibroblasts and smooth muscle cells to proliferate and migrate into the wound site [34]. *In vitro*, PGDF stimulates fibroblasts to contract collagen matrices and differentiate into myofibroblasts [35].

Studies have revealed that PDGF levels are elevated in the bronchial lavage fluid of SSc patients, as well as elevated levels of the PDGFβ receptors on SSc fibroblasts [36-38]. Moreover, one study has been reported showing that autoantibodies stimulating the PDGF receptor may be a hallmark of SSc [39].

Mice treated with PDGFβ receptor- inhibitor imatinib mesylate, a tyrosine kinase inhibitor exhibit delayed cutaneous wound closure, diminished numbers of myofibroblast numbers and reduced collagen type I expression [40]. Imatinib mesylate did not prevent the myofibroblast differentiation *in vitro *but inhibited fibroblast proliferation and migration and appeared to principally act by blocking pericyte recruitment [40] (Figure 1). As a subset (~30%) of myofibroblasts in cutaneous mouse wounds are NG2-positive pericytes, this phenomenon is likely to lead to the reduction myofibroblasts in the wound [41]. Intriguingly, however, the majority (~70%) of myofibroblasts in bleomycin-induced skin fibrosis are derived from pericytes [42]. Tyrosine kinase inhibitors analogous to imatinib mesylate blocked bleomycin-induced dermal fibrosis in mice [43]. It is also interesting to note that imatinib mesylate also blocks the ability of TGFβ to activate Smad 1 and the transcription factor egr-1 via c-abl, emphasizing the potential of signalling crosstalk between PDGF and non-canonical TGFβ signalling and further suggesting that this inhibitor may also work by blocking non-canonical TGFβ signalling [44,45]. Given that pericytes contribute to myofibroblast activation in SSc [46], these results collectively suggest that perhaps targeting PDGF/c-abl might be of benefit in SSc through its ability to block pericyte recruitment. As such, anti-PDGF drugs may represent a different sort of approach to alleviating SSc than blocking growth factor differentiation of resident fibroblasts, which may be of lesser importance than pericyte recruitment in generating a source of myofibroblasts in fibrosis.

## Future Prospects and Conclusions

TGFβ, ET-1, CCN2 and PDGF are likely to cooperate in driving tissue repair and fibrogenic responses in lesional SSc fibroblasts. However, these proteins seem to be responsible, for somewhat differing activities suggesting that combination therapies may be appropriate for SSc.

## Abbreviations

α -SMA: α-smooth muscle actin; CTGF: connective tissue growth factor; ECM: extracellular matrix; ERK: extracellular signal-regulated kinase-1; ET: endothelin; FAK: focal adhesion kinase; HSPG: heparan sulphate-containing proteoglycan; PDGF: platelet derived growth factor; SSc: systemic sclerosis; TGFβ: transforming growth factor β; TAK: TGFβ activated kinase 1

## Competing interests

The author declares that they have no competing interests.
